# A Fully-Human Antibody Specifically Targeting a Membrane-Bound Fragment of CADM1 Potentiates the T Cell-Mediated Death of Human Small-Cell Lung Cancer Cells

**DOI:** 10.3390/ijms23136895

**Published:** 2022-06-21

**Authors:** Ji Hyun Lee, Ji Woong Kim, Ha Rim Yang, Seong-Won Song, Song-Jae Lee, Yeongha Jeon, Anna Ju, Narim Lee, Min-Gu Kim, Minjoo Kim, Kyusang Hwang, Jin Hwan Yoon, Hyunbo Shim, Sukmook Lee

**Affiliations:** 1 Department of Biopharmaceutical Chemistry, Kookmin University, Seoul 02707, Korea; 707jh@kookmin.ac.kr (J.H.L.); 2825760@kookmin.ac.kr (H.R.Y.); kyusang@kookmin.ac.kr (K.H.); yoonjinhwan8090@kookmin.ac.kr (J.H.Y.); 2 Department of Chemistry, Kookmin University, Seoul 02707, Korea; jwk7853@kookmin.ac.kr; 3 R&D Center, CellabMED Inc., 161, Jeongneung-ro, Seongbuk-gu, Seoul 02708, Korea; swsong@cellabmed.com (S.-W.S.); sjlee@cellabmed.com (S.-J.L.); yeongha0820@cellabmed.com (Y.J.); anna@cellabmed.com (A.J.); narim.lee@cellabmed.com (N.L.); alsrn0520@cellabmed.com (M.-G.K.); delijoo@cellabmed.com (M.K.); 4 Department of Life Science, Ewha Womans University, Seoul 03760, Korea; hshim@ewha.ac.kr; 5 Biopharmaceutical Chemistry Major, School of Applied Chemistry, Kookmin University, Seoul 02707, Korea; 6 Antibody Research Institute, Kookmin University, Seoul 02707, Korea

**Keywords:** fully human antibody, MF-CADM1, cell death, T cell, small cell lung cancer

## Abstract

Small-cell lung cancer (SCLC) is the most aggressive form of lung cancer and the leading cause of global cancer-related mortality. Despite the earlier identification of membrane-proximal cleavage of cell adhesion molecule 1 (CADM1) in cancers, the role of the membrane-bound fragment of CAMD1 (MF-CADM1) is yet to be clearly identified. In this study, we first isolated MF-CADM1-specific fully human single-chain variable fragments (scFvs) from the human synthetic scFv antibody library using the phage display technology. Following the selected scFv conversion to human immunoglobulin G1 (IgG1) scFv-Fc antibodies (K103.1–4), multiple characterization studies, including antibody cross-species reactivity, purity, production yield, and binding affinity, were verified. Finally, via intensive in vitro efficacy and toxicity evaluation studies, we identified K103.3 as a lead antibody that potently promotes the death of human SCLC cell lines, including NCI-H69, NCI-H146, and NCI-H187, by activated Jurkat T cells without severe endothelial toxicity. Taken together, these findings suggest that antibody-based targeting of MF-CADM1 may be an effective strategy to potentiate T cell-mediated SCLC death, and MF-CADM1 may be a novel potential therapeutic target in SCLC for antibody therapy.

## 1. Introduction

Lung cancer is the leading cause of cancer-related mortality worldwide [1]. More specifically, an estimated 2,206,771 new cases and 1,796,144 deaths from lung cancer were reported globally in 2020 [2]. Lung cancers include non–small-cell lung cancer (NSCLC) and small-cell lung cancer (SCLC) [3]. Especially, SCLC is a highly aggressive neuroendocrine carcinoma that represents approximately 10%–15% of lung cancers with an exceptionally poor prognosis [4]. Furthermore, SCLC tends to grow and spread faster than NSCLC [5]. In the United States (US), approximately 30,000–35,000 people are diagnosed with SCLC each year [6]. Currently, SCLC has two stages, namely, the limited and extensive stages [7]. Limited-stage SCLC is only present in one lung and potentially in nearby lymph nodes to the same side of the chest, whereas extensive-stage SCLC spreads to the opposite side of the chest or distant organs [8]. Over several decades, a variety of therapeutic regimens, including chemotherapy, radiation therapy, immunotherapy, surgery, and combination therapy, have been clinically used for SCLC treatment [9]. These treatment options are mainly determined based on the stage of cancer, but other factors, such as a person’s overall health and lung function, are also considered [10]. However, the 5-year survival rate for SCLC is reported to be relatively low (6.5%) [11].

Monoclonal antibody (mAb) is a laboratory-produced molecule that is engineered to study disease-related molecular mechanisms and/or treat various diseases, such as infectious diseases, immunological disorders, and cancers [12,13]. Currently, mAb therapy is one of the most effective treatments for cancers [14]. Since the first approval of the US Food and Drug Administration (FDA) for OKT3, a mouse anti-CD3 mAb, 131 therapeutic antibodies have been approved by the US FDA and/or European Medicines Agency (EMA) thus far [15,16]. Among them, 59 are indicated for cancer treatment. Some immune checkpoint inhibitors, such as atezolizumab, which is a humanized IgG1 antibody to PD-L1, and durvalumab, which is a fully human immunoglobulin G1 (IgG1) antibody to PD-L1, are currently used for SCLC treatment in combination with chemotherapy [17]; however, according to a phase III clinical trial results, their therapeutic efficacy is not dramatic [18]. In detail, the median overall survival (OS) of atezolizumab plus carboplatin and the etoposide-treated group was 12.3 months, which was 2 months longer than that of the carboplatin and etoposide-treated groups (10.3 months) [19]. Furthermore, the median OS of durvalumab plus platinum-etoposide-treated groups (13 months) was 2.7 months longer than that of the platinum-etoposide-treated group (10.3 months) [20]. Therefore, identifying the novel potential therapeutic targets in SCLC is essential for not only better understanding their functional role and relevance in SCLC but also for developing novel therapeutics for improving the clinical outcomes of patients with SCLC.

Cell adhesion molecule 1 (CADM1), also known as IGSF4, TSLC1, Necl-2, and SynCAM1, is a member of the Immunoglobulin (Ig) superfamily that consists of an extracellular domain (ECD) containing three Ig-like loops, a single transmembrane domain (TM), and a cytoplasmic domain (CD) [21]. CADM1 protein is expressed in some normal tissues, including epithelial, neuronal, lung, brain, pancreas, and testis tissues [22,23,24,25]. It plays a vital role in the regulation of cell adhesion, migration, and survival [22,26,27]. CADM1 seems to play different roles depending on the cancer type. Many studies reported CADM1 as a tumor suppressor in several cancer types, including ovarian, breast, and pancreatic cancers, and the loss of CADM1 expression is closely associated with cancer progression and metastasis [28,29,30]. Contrarily, CADM1 overexpression in SCLC is particularly associated with tumorigenicity, suggesting its unique oncogenic role in SCLC [31,32]. Furthermore, CADM1, encoded by 12 exons, undergoes alternative splicing to generate several splicing variants through combining alternative exons 8/9/10. Among them, variants 8 and 8/9 of CADM1 are almost exclusively observed in SCLCs [32]. Variant 8/9 is susceptible to cleavage by proteases, such as a disintegrin and metalloprotease 10 (ADAM10), and γ-secretases, and it generates the membrane-bound fragment of CADM1 (MF-CADM1) on the cancer cell surface, whereas variant 8 was known to be a cleavage-resistant form of CADM1 [33]. In addition, normal lung and brain tissues did not express variant 8/9; however, its expression could be detected in testis, suggesting that the expression of MF-CADM1 might be specific to SCLC [32]. However, the role of MF-CADM1 in SCLC has not been clearly identified yet.

Therefore, for the first time, this study developed fully human antibodies that are specific to human and mouse MF-CADM1 using a phage display technology. Our intensive in vitro characterization and functional analyses with these antibodies provided a proof of concept that antibody-based targeting of MF-CADM1 may potentiate the T cell-mediated cell death of SCLCs and that MF-CADM1 may be a novel potential therapeutic target in SCLCs for antibody therapy.

## 2. Results

### 2.1. Preparation and Analysis of MF-CADM1 Peptide Conjugates for Antibody Selection

**Figure 1:**
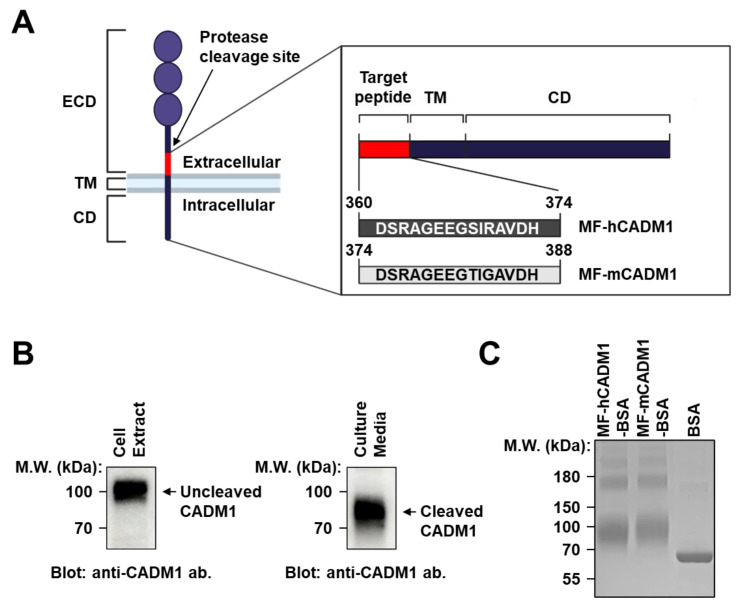
Cleavage of CADM1 protein and preparation of CADM1 peptide conjugates as antigens for antibody selection. (**A**) Schematic drawing of the membrane-proximal cleavage of CADM1 and its resulting membrane-bound fragment of CADM1 (MF-CADM1) on cancer cell surfaces. (**B**) NCI-H69 SCLC cell extracts (left) or the culture media (right) were individually subjected to immunoblot analysis with commercially available anti-CADM1 antibodies. Beta-actin was used as a loading control. (**C**) MF-hCADM1-BSA, MF-hCADM1-BSA, or BSA as a negative control, was subjected to SDS-PAGE, and the mobility shift was visualized by Coomassie Brilliant Blue (CBB) staining.

CADM1 is an Ig superfamily member that promotes the malignant features of SCLCs [31]. Reportedly, some proteases, such as ADAM10 and γ-secretase, induce the membrane-proximal cleavage of CADM1, called shedding, that results in MF-CADM1 formation on tumor cells (Figure 1A) [33]. To investigate whether the cleavage of CADM1 also occurs on NCI-H69 human SCLC cells, the cell extracts and culture media were individually collected and subjected to immunoblot analysis with a commercially available anti-CADM1 antibody that is specific to the second Ig-like domain in the ECD of CADM1. We detected a discrete CADM1 band that had a smaller molecular weight in the media than that in the cell extract, thereby suggesting the membrane-proximal cleavage of CADM1 on the NCI-H69 cells (Figure 1B).

To generate MF-CADM1 peptide conjugates as antigens for antibody selection, we first synthesized the peptides of human MF-CADM1 (MF-hCADM1; amino acids 360–374) or mouse MF-CADM1 (MF-mCADM1; amino acids 374–388), respectively, and conjugated them to bovine serum albumin (BSA) as a carrier protein (MF-hCADM1-BSA and MF-mCADM1-BSA). The quality of the MF-CADM1 peptide conjugates was then assessed with sodium dodecyl sulfate-polyacrylamide gel electrophoresis (SDS-PAGE) followed by Coomassie Brilliant Blue (CBB) staining. We confirmed the mobility shift of MF-hCADM1-BSA or MF-mCADM1-BSA compared with BSA as a negative control, showing the proper conjugation of MF-CADM1 peptide conjugates (Figure 1C). These conjugates were then used for antibody selection using phage display technology.

### 2.2. Selection of Fully Human scFvs Specific to Human and Mouse MF-CADM1

**Figure 2:**
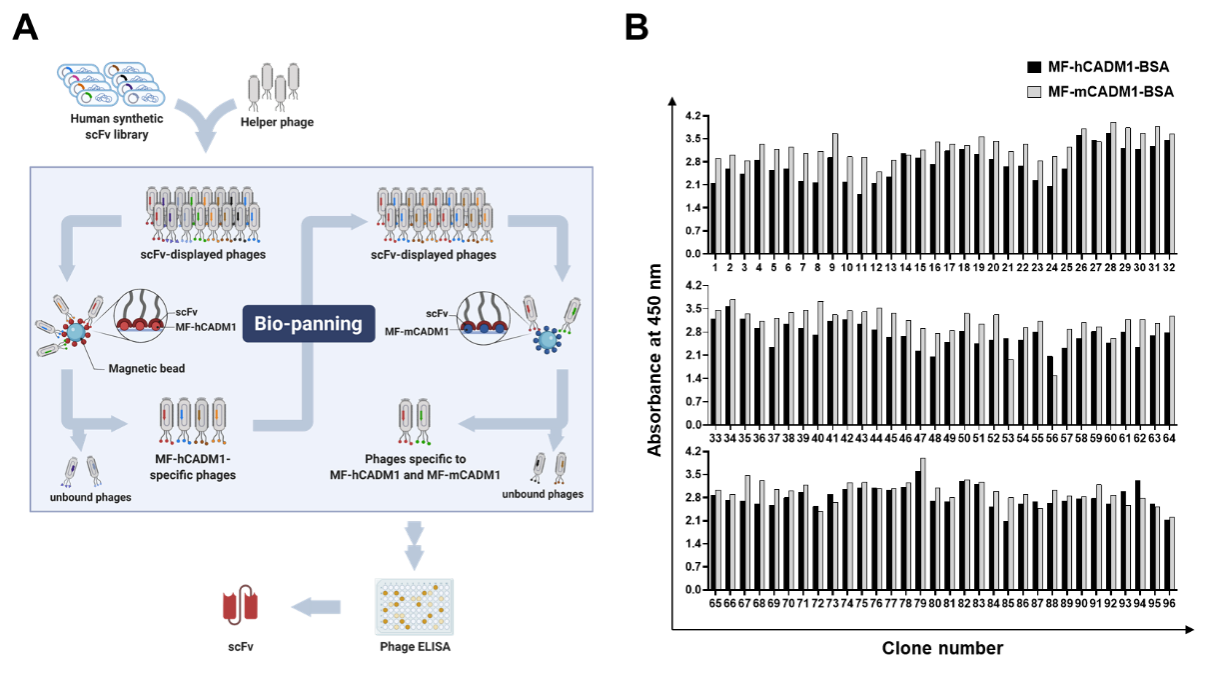
Selection of human and mouse MF-CADM1-specific scFv clones using phage display technology. (**A**) Schematic representation of the scFv antibody selection procedures using phage display bio-panning. Six rounds of bio-panning were performed with the MF-hCADM1- or MF-mCADM1-BSA-immobilized magnetic beads. (**B**) Phage ELISA, with 96 randomly selected phage clones, was performed to select MF-hCADM1- and MF-mCADM1-specific scFv clones.

To select fully-human antibodies having cross-species reactivity to human and mouse MF-CADM1, we performed phage display bio-panning with the MF-hCADM1- or MF-mCADM1-BSA-immobilized magnetic beads and selected scFv clones specific to both MF-hCADM1 and MF-mCADM1 from a human synthetic scFv antibody library (Figure 2A). In detail, following the consecutive six rounds of bio-panning by increasing detergent concentration and washing frequency, 96 clones from output titer plates were randomly selected, rescued by the helper phages, and tested for their reactivity to MF-hCADM1 and MF-mCADM1 using phage enzyme-linked immunosorbent assay (phage ELISA). All of the selected scFv clones were found to have strong reactivity to MF-hCADM1- and MF-mCADM1-BSA (Figure 2B), but not to BSA, as a negative control (data not shown), showing the specific binding of the selected scFvs to MF-hCADM1 and MF-mCADM1. Finally, the deoxyribonucleic acid (DNA) sequencing results reveal that four scFv clones have different complementarity-determining region (CDR) sequences (data not shown).

### 2.3. Biophysiochemical Characterization of the Selected scFv-Fc Antibodies

**Figure 3:**
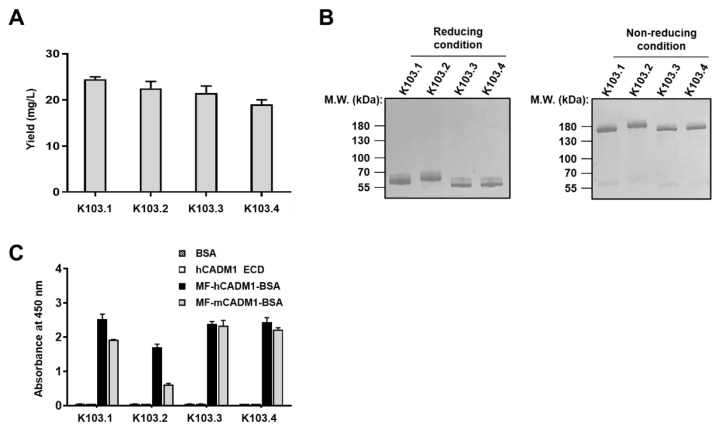
Biochemical characterization of the four selected scFv-Fc antibodies. (**A**) The selected antibodies were transiently overproduced and purified using affinity column chromatography. The production yield of each scFv-Fc antibody was shown as graph bars. (**B**) The conformation status of the scFv-Fcs was confirmed by SDS-PAGE under reducing and non-reducing conditions, followed by CBB staining. (**C**) ELISA was performed with recombinant human CADM1-ECD, MF-hCADM1-BSA, or MF-mCADM1-BSA to verify the specific binding of each antibody to MF-CADM1. BSA alone was used as a negative control.

Following the conversion of the selected scFvs to human IgG1 scFv-Fc antibodies, the antibodies were transiently overproduced in Expi293F cells and purified using affinity column chromatography with protein A sepharose, respectively. Here, we designated four scFv-Fc antibodies as K103.1–4 and found that all the scFv-Fc antibodies have a production yield of approximately 20 mg/L (Figure 3A). Further, the endotoxin contamination in each antibody preparation was individually tested, wherein negligible endotoxin was detected (data not shown).

To examine the conformation status of the scFv-Fcs, each scFv-Fc clone, in the presence or absence of dithiothreitol as a reducing agent, was subjected to SDS-PAGE followed by CBB staining. We observed that each clone is monomeric in the reducing condition, whereas it has dimeric a conformation as expected in the non-reducing condition (Figure 3B).

To verify the specific binding of each scFv-Fc antibody to human and mouse MF-CADM1, we individually performed ELISA with recombinant human CAMD1-ECD, MF-hCAMD1-BSA, or MF-mCADM1-BSA. Here, BSA is used as a negative control. We found that all the selected scFv-Fc antibodies bind to MF-hCADM1- and MF-mCADM1-BSA but not to recombinant human CAMD1-ECD or BSA alone (Figure 3C).

**Figure 4:**
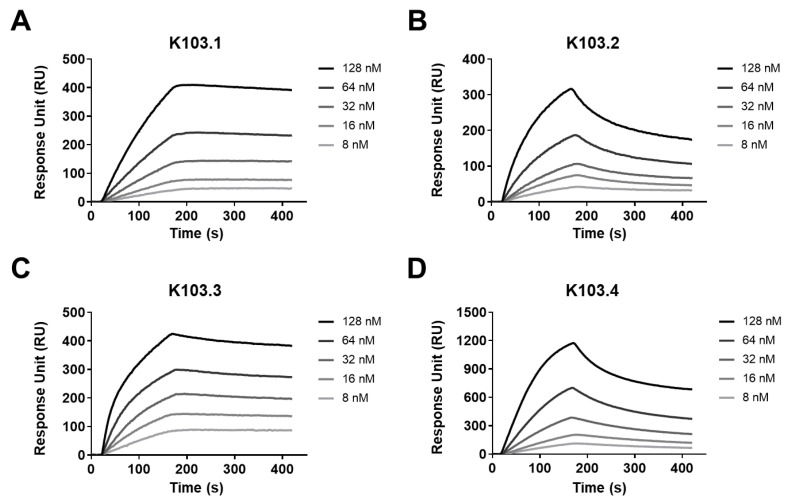
Kinetic analysis of the interaction between the selected scFv-Fc antibodies and MF-hCADM1. The binding affinities of the selected antibodies to MF-hCADM1 were measured using SPR. Each SPR sensorgram shows the interaction of the different concentrations (8, 16, 32, 64, and 128 nM) of (**A**) K103.1, (**B**) K103.2, (**C**) K103.3, and (**D**) K103.4 to an MF-hCADM1-BSA-immobilized sensor chip.

To investigate the binding affinity of each scFv-Fc antibody to human MF-CADM1, we performed surface plasmon resonance (SPR). In detail, following the immobilization of MF-hCAMD1-BSA onto a sensor chip, the binding kinetics of antigen-antibody interaction was measured by increasing the concentration of the selected scFv-Fc antibodies (Figure 4A–D). We demonstrated that K103.1, K103.2, K103.3, and K103.4 specifically bind to MF-hCADM1 with a dissociation constant (K_D_) of ~4.5, 39, 2.3, and 41 nM, respectively (Table 1).

### 2.4. Effect of the Selected scFv-Fc Antibodies on Jurkat T Cell-Mediated SCLC Cell Death

**Figure 5:**
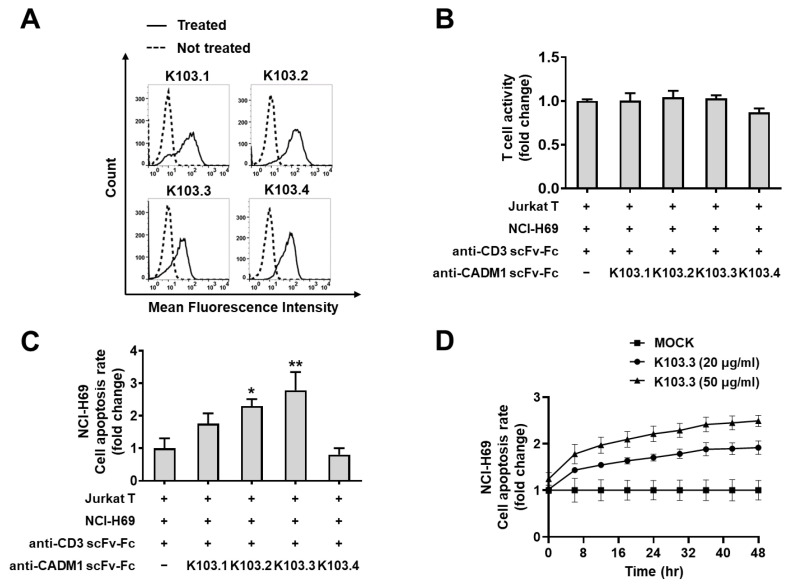
Effect of the selected scFv-Fc antibodies on death in NCI-H69 cells by activated Jurkat T cells. (**A**) The binding of the selected antibodies to MF-CADM1 on NCI-H69 cells was investigated in the presence (solid line) or absence (dashed line) of the selected antibodies using flow cytometry. (**B**) T cell activity was compared by measuring the fluorescence of GFP expressed in activated Jurkat T cells in the presence or absence of selected antibodies. (**C**) The death rate of NCI-H69 cells using the activated Jurkat T cells in the presence or absence of the selected antibodies, respectively. (**D**) Antibody concentration-dependent death in NCI-H69 cells was measured. All values represent the mean ± SEM of triplicate measurements from one of three independent experiments. * *p* < 0.05; ** *p* < 0.01.

To examine the surface binding of each scFv-Fc antibody to MF-CADM1 on SCLC cells, we conducted flow cytometry with each scFv-Fc and NCI-H69 cell. The result revealed that all the antibodies bind to the NCI-H69 cell surface (Figure 5A), whereas control IgG has no binding (Supplementary Figure S1), indicating the specific interaction of the antibodies to the considerable amount of MF-CADM1 on NCI-H69 cells. To further test the specific binding of the antibody to MF-CADM1 on NCI-H69 cells, we pre-incubated K103.3 in the presence or absence of MF-hCADM1 peptide-conjugated BSA and then conducted flow cytometry. We found that pre-incubation of K103.3 with MF-hCADM1 reduces the antibody binding to the NCI-H69 cell surface, indicating the specific binding of K103.3 on MF-CADM1 on the cells (Supplementary Figure S2).

To investigate the role of the selected antibodies on T cell-mediated death in SCLC cells, we first generated an antibody specific to CD3ε for T cell activation and prepared engineered Jurkat T cells that specifically express a green fluorescent protein (GFP) upon T cell activation. Then, we treated NCI-H69 with this anti-CD3ε antibody in the presence or absence of each scFv-Fc antibody and measured the T cell activity. We confirmed that the selected antibodies do not affect the Jurkat T cell activation induced by the anti- CD3ε antibody (Figure 5B). Simultaneously, we incubated NCI-H69 cells with IncuCyte annexin V red reagent, a fluorescent death dye, and quantified the extent of NCI-H69 cell death in the presence or absence of each scFv-Fc antibody in the same experimental settings. We found that, among the selected antibodies, K103.3 most potently induces the death of NCI-H69 cells by the activated Jurkat cells (Figure 5C). Further, we detected that K103.3-induced death in NCI-H69 cells was increased in an antibody concentration-dependent manner (Figure 5D).

**Figure 6:**
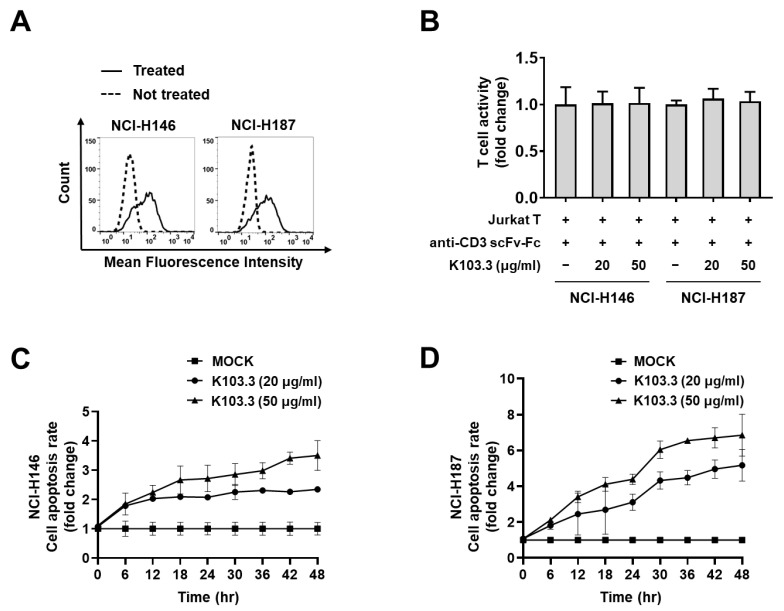
Effect of the selected scFv-Fc antibodies on death in NCI-H146 and NCI-H187 cells by activated Jurkat T cells. (**A**) The binding of the selected antibodies to MF-CADM1 on NCI-H146 and NCI-H187 cells were examined in the presence (solid line) or absence (dashed line) of the selected antibodies using flow cytometry. (**B**) T cell activity was compared by measuring the fluorescence of GFP expressed in activated Jurkat T cells in the presence or absence of selected antibodies. Antibody concentration-dependent death in NCI-H146 (**C**) and NCI-H187 cells (**D**) in the presence or absence of the selected antibodies was measured. All values represent the mean ± SEM of triplicate measurements from one of three independent experiments.

To further examine the universal effect of K103.3 on T cell-mediated death in SCLC cells, we also performed flow cytometry with K103.3 and other SCLC cell lines, such as NCI-H146 and NCI-H187. We confirmed the binding of K103.3 on the surface of both NCI-H146 and NCI-H187 cells (Figure 6A). Then, we incubated the activated Jurkat T cell with NCI-H146 or NCI-H187 cells in the presence or absence of the K103.3 antibody. We confirmed that the selected antibodies did not affect the Jurkat T cell activation induced by the anti-CD3ε antibody (Figure 6B). Simultaneously, we found that K103.3 increases the Jurkat T cell-mediated death in NCI-H146 (Figure 6C) or NCI-H187 (Figure 6D) in an antibody concentration-dependent manner.

### 2.5. Effect of K103.3 on Endothelial Cell Toxicity

**Figure 7:**
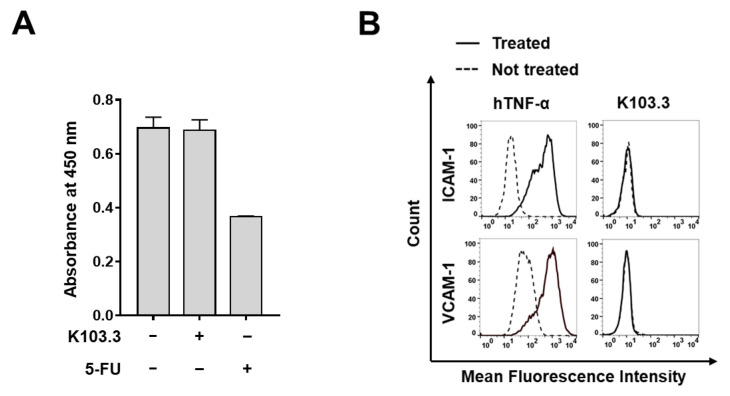
Effect of K103.3 on endothelial cell toxicity. (**A**) HUVECs were incubated in the absence or presence of K103.3 or 5-FU. Cell viability was measured at an absorbance of 450 nm. Values represent the mean ± SEM of triplicate measurements. (**B**) HUVECs were cultured in the presence or absence of hTNF-α or K103.3, stained with anti-ICAM-1 (**upper** panel) or VCAM-1 (**lower** panel) polyclonal antibody, and analyzed using flow cytometry.

To investigate the effect of K103.3 on endothelial cell viability, we first evaluated the viability of human umbilical vein endothelial cells (HUVECs) in the absence or presence of K103.3 and 5-fluorouracil (5-FU) as a positive control. We confirmed that K103.3 did not affect HUVEC viability, whereas 5-FU significantly reduced the cell viability (Figure 7A). Furthermore, we treated HUVECs with K103.3 or human tumor necrosis factor α (hTNFα) as a positive control. We monitored the HUVEC activation by measuring the expression of vascular cell adhesion molecule-1 (VCAM-1) and intracellular cell adhesion molecule-1 (ICAM-1), endothelial cell activation markers. We found that K103.3 did not affect the HUVEC activation, while hTNFα significantly induced the cell activation (Figure 7B). Additionally, to investigate the possible adverse effects of the antibody in normal cells, we first evaluated the MF-CADM1 expression on HUVECs using flow cytometry. The result showed that CADM1, but not MF-CADM1, is expressed on HUVECs (Supplementary Figure S4). Next, we incubated the activated Jurkat T cell with HUVECs in the presence or absence of the K103.3 antibody. We confirmed that the selected antibody did not induce Jurkat T cell-mediated death in HUVECs in an antibody concentration-dependent manner (Supplementary Figure S5).

## 3. Discussion

SCLC is an aggressive form of lung cancer that has metastasized to other body areas by the time it is diagnosed [34]. To date, some immunotherapy treatments have been developed for the treatment of patients with SCLCs [35]. The two US FDA-approved immune checkpoint inhibitors, namely, durvalumab and atezolizumab, are clinically used; however, these have shown limited clinical efficacy because the median OS is only extended by < 3 months [19,20]. Furthermore, other than these inhibitors, there are no other options for antibody therapy for SCLC treatment [17]. Therefore, continuous efforts are needed to identify promising potential therapeutic targets in SCLCs and therapeutics to improve the clinical outcome of patients with SCLCs. In this study, we selected MF-CADM1-specific fully human antibodies, a cleaved fragment of CADM1 on the cell surface of advanced SCLCs, using phage display technology. Through intensive characterization and functional studies, we demonstrated that an antibody targeting MF-CADM1 shows promising anti-tumor activity by promoting the T-cell-mediated cell death of SCLCs. Here, based on our findings, we suggest that MF-CADM1 may be a novel potential therapeutic target in SCLCs, and antibody-based targeting of MF-CADM1 may be effective against SCLC.

Antibody therapy is one of the targeted therapies that specifically modulates a causative factor in cancer and blocks a specific signaling pathway that is closely associated with cancer progression and metastasis [36,37]. As well known, the target specificity of antibodies leads to fewer adverse effects and superior efficacy and/or in combination with pre-existing chemotherapeutic agents in cancer therapy [38]. Based on the present study findings, we propose that the anti-MF-CADM1-specific antibodies that we have developed can be used as antibody platforms for a wide range of use, such as research and pharmaceutical purposes. Several lines of evidence support our hypothesis. First, K101.3 seems to be specific to MF-CADM1. Our phage ELISA and ELISA with the selected scFv-Fc antibodies (K103.1~4) show that all scFv or scFv-Fc clones specifically recognize MF-CADM1-BSA but not BSA. All scFv-Fcs only bind to MF-CADM1-BSA but not to rhCADM1-ECD, and BSA further supports the specific binding of the antibodies to MF-CADM1. Furthermore, we confirmed that K103.3 does bind to MF-CADM1-KLH but not to KLH alone (Supplementary Figure S1). Second, all scFvs that are isolated from the human synthetic antibody library are fully human antibodies. Thus, they may relatively lead to lower immunogenicity than the mouse or chimeric antibodies in vivo. Third, it has broad cross-species reactivity to human and mouse MF-CADM1 that can apply to preclinical and clinical safety and efficacy evaluation studies. In phage ELISA and ELISA with selected antibodies, we observed that all the scFvs or scFv-Fcs specifically recognize both MF-hCADM1-BSA and MF-mCADM1-BSA, but not BSA. Fourth, our SPR analysis showed that K103.3 has a high affinity (K_D_ approximately 2.3 nM) to MF-hCADM1, indicating strong binding toward the target antigen. Fifth, K103.3 specifically target MF-CADM1 expression on the membrane surface of SCLC cells. Using flow cytometry, K103.3 specifically binds to a broad range of SCLC cell lines, including NCI-H69, NCI-H146, and NCI-H526, whereas control scFv-Fc does not bind to these cell lines (Supplementary Figure S2). Furthermore, K103.3, pre-incubated with MF-hCADM1, does not bind to these SCLC cell lines (Supplementary Figure S3). Lastly, we confirmed that all the selected scFv-Fc antibodies do not form visible aggregates during antibody purification, indicating that the antibodies are physiochemically stable. Taken together, these results suggest that the anti-MF-CADM1-specific antibodies may be useful not only for identifying the functional role and relevance of MF-CADM1 in SCLCs but also for developing the next-generation SCLC therapeutics for antibody therapy.

Cancer immunotherapy is a type of cancer treatment that boosts the body’s immune system to combat cancer [39]. Upon the activation of T cells, it plays a pivotal role in cancer cell death by secreting a variety of factors, including perforin, granzymes, and cytokines [40]. By monitoring cell death using IncuCyte annexin V red reagent in the present study, we found that K103.3 significantly promotes the death of three types of SCLC cell lines, such as NCI-H69, NCI-H146, and NCI-H187 cells, by the activated Jurkat T cells without interfering with the T cell activation. Furthermore, we found that each cancer cell death was increased in a K103.3 antibody concentration-dependent manner. This evidence leads us to speculate that K103.3 may act as a facilitator to recruit the activated T cells near MF-CADM1-expressing SCLC cells and result in efficient SCLC cell death. In these functional analyses, we have used an engineered Jurkat T cell line as a model cell line to simultaneously monitor T cell activation and cancer cell death. This study has focused on identifying the role of K103.3 on SCLC cell death by activated Jurkat T cells; however, we cannot exclude the possibility that CD8-positive cytotoxic T cells participate in T cell-mediated cell death of SCLCs in vivo. Additionally, through our in vitro toxicity evaluation studies, we demonstrated that K103.3 does not affect the HUVEC activation, as well as the HUVEC viability, suggesting that this antibody may not induce significant endothelial cell toxicity in vivo. Taken together, although further in vivo studies are needed, based on our findings, we suggest that MF-CADM1 may be a novel potential therapeutic target in SCLCs and that antibody-based targeting of MF-CADM1 may be effective against SCLCs.

In conclusion, based on currently available evidence, we suggest a mode of action whereby MF-CADM1-specific IgG-based antibody can promote the efficient recruitment of T cells by interactions between the Fc region of the antibody and Fc gamma receptors expressing on T cells and redirect the activated T cells proximal to the membrane surface of MF-CADM1-expressing SCLC cells, resulting in potentiating T cell-mediated SCLC cell death. Furthermore, despite our antibodies being developed as mAbs, these antibodies are also used as antibody platforms to generate bi- or multi-specific immune cell engagers or chimeric antigen receptor T cells for the effective treatment of patients with SCLCs. In future studies, we plan to further investigate the action mechanism in more detail and evaluate in vivo efficacy, in combination with immune checkpoint inhibitors and against MF-CADM1-expressing SCLCs.

## 4. Materials and Methods

### 4.1. Cell Culture

NCI-H69, NCI-H146, and NCI-H187 human SCLC cells (Korean Cell Bank, Seoul, Korea), and NF-κB/Jurkat/GFP™ transcriptional reporter cells (System Bioscience, Palo alto, CA, USA) were cultured in RPMI 1640 medium (Gibco, Waltham, MA, USA) supplemented with 10% (*v*/*v*) fetal bovine serum (FBS; Gibco) and 1% (*v*/*v*) penicillin/streptomycin (Gibco) and maintained at 37 °C with 5% CO_2_. HUVECs were cultured in Endothelial Cell Growth Medium-2 (EGM-2) (Lonza, Basel, Switzerland) at 37 °C with 5% CO_2_. The Expi293F cells (Gibco) were cultured in Expi293 expression medium (Gibco) in a humidified shaking incubator at 37 °C with 8% CO_2_.

### 4.2. Immunoblot Analysis

The NCI-H69 cells were lysed with RIPA buffer (Thermo Fisher Scientific, Waltham, MA, USA) supplemented with a protease inhibitor cocktail (GenDEPOT, Katy, TX, USA). NCI-H69 cultured media was concentrated with Amicon^®^ Ultra Centrifugal Filters (Merck Millipore, Burlington, MA, USA) following the manufacturer’s protocol. Then, the amount of total protein was determined using the BCA assay kit (Thermo Fisher Scientific, Waltham, MA, USA). Then, 10 μg of protein samples in the cell extracts or cultured media were resolved by SDS-PAGE and transferred to nitrocellulose membrane (GE Healthcare, Chicago, IL, USA). The membrane was incubated with chicken anti-CADM1 mAb (1:1000; MBL, Tokyo, Japan) or mouse anti-β-actin antibody (1:3000; Santa Cruz Biotechnology, Dallas, TX, USA), respectively, overnight at 4 °C. After several washes with tris-buffered saline with 0.1% (*v*/*v*) tween 20 (TBST), the membranes were incubated with horseradish peroxidase (HRP)-conjugated anti-chicken IgY antibody (1:5000; Abcam, Cambridge, UK), or HRP-conjugated anti-mouse IgG antibody (Seracare Life Science, Milford, MA, USA) for 1 h at room temperature. After washing with TBST buffer three times, the protein bands were visualized using SuperSignal West Pico PLUS Chemiluminescent Substrate (Thermo Fisher Scientific, Waltham, MA, USA) following the manufacturer’s instructions.

### 4.3. Analysis of MF-CADM1 Peptide-Conjugates and Purified Antibodies

MF-hCADM1 or MF-mCADM1 peptide-conjugated BSA was generated by peptide synthesis (Peptron, Daejeon, Korea). Then, 2 μg of MF-hCADM1 or MF-mCADM1 peptide-conjugated BSA or BSA were only resolved by SDS-PAGE with a 12% polyacrylamide under reducing conditions. The same amount of selected scFv-Fc antibodies was separated under reducing or non-reducing conditions. After separation, each band on the resulting gels was visualized by CBB staining.

### 4.4. Isolation of Fully Human scFv Antibodies Specific to MF-hCADM1 and MF-mCADM1

Phage display bio-panning was conducted to select MF-hCADM1- and MF-mCADM1-specific fully human scFv antibodies from a human synthetic scFv antibody library. In detail, 4 μg of MF-hCADM1 or MF-mCADM1 peptide-conjugated BSA for each bio-panning was first immobilized onto magnetic beads (Dynabeads M-270 epoxy, Invitrogen™, Waltham, MA, USA). Then, to select scFv clones cross-reactive to human and mouse MF-CADM1, six rounds of bio-panning were consecutively performed by alternately using magnetic beads immobilized with MF-hCADM1 peptide conjugate for the first, third, and fifth bio-panning or MF-mCADM1 peptide conjugate for the second, fourth, and sixth bio-panning. Finally, 96 phage clones were randomly selected from output colonies and tested for their reactivity to target antigens by phage ELISA. Following DNA sequencing, scFv clones with different CDR sequences were selected and used for the next studies.

### 4.5. Phage ELISA

Single colonies were inoculated into 1 mL of SB media containing 50 μg/mL of carbenicillin in 96-deep-well plates (Axygen, Union City, CA, USA) and incubated at 37 °C overnight. Then, 10^12^ pfu/mL helper phages (VCSM13; Agilent, Santa Clara, CA, USA) and 70 μg/mL of kanamycin were added and incubated overnight at 37 °C. The plates were centrifuged at 4000 rpm, and the supernatant was applied for phage ELISA. The 96-well high-binding microplates (Corning, Corning, NY, USA) were coated overnight with 0.1 μg of MF-hCADM1 and MF-mCADM1 in phosphate-buffered saline (PBS) at 4 °C. After blocking with 3% (*w*/*v*) BSA in PBS, the plates were incubated with 100 μL of phage supernatant at 37 °C for 2 h. After washing three times with PBS containing 0.05% (*v*/*v*) tween 20 (PBST), HRP-conjugated anti-hemagglutinin (HA) antibody (1:3000; Bethyl Laboratories, Montgomery, TX, US) was added and incubated at 37 °C for 1 h. Colorimetric detection was performed by adding 3,3′,5,5′-tetramethylbenzidine (TMB) substrate solution (Thermo Fisher Scientific, Waltham, MA, USA) as the chromogenic substrate. The reactions were stopped by the addition of 2 M of sulfuric acid (H_2_SO_4_), and the absorbance was read at 450 nm using a microtiter plate reader (Bio-Tek Instruments, Winooski, VT, USA).

### 4.6. Generation of scFv-Fc Antibodies

To generate the scFv-Fc forms of antibodies, the selected scFv clones were individually subcloned into the pCEP4 mammalian expression vector containing the fragment crystallizable (Fc) region of human IgG1. The recombinant vectors that encode the scFv-Fc antibodies were transfected into Expi293 cells using the Expi293 transfection kit (Thermo Fisher Scientific, Waltham, MA, USA) following the manufacturer’s instruction and cultured at 37 °C with shaking at 125 rpm and 8% CO_2_. On the 7th day after transfection, the resulting supernatants were collected for antibody purification using affinity chromatography with protein A sepharose (GE Healthcare, Chicago, IL, USA) as previously described [41].

### 4.7. ELISA

Then, 0.05 μg of MF-hCADM1 or MF-mCADM1 peptide-conjugated BSA were coated on 96-well plates (Corning) overnight at 4 °C. After blocking with 3% BSA in PBS, the plates were incubated with the diluted antibodies in blocking buffer at 37 °C for 2 h. Following several washes with PBST, the plates were then incubated with HRP-conjugated anti-HA antibody (1:5000; Bethyl Laboratories) at 37 °C for 1 h. Finally, 100 μL of TMB substrate (Thermo Fisher Scientific, Waltham, MA, USA) to each well was added for colorimetric detection, and the reaction was stopped with the addition of 2N H_2_SO_4_ solution. The absorbance was measured at 450 nm using a microtiter plate reader (Bio-Tek Instruments, Winooski, VT, USA).

### 4.8. SPR

The real-time interaction of antibody and antigen was measured at room temperature using SPR with an iMSPR mini-instrument (Icluebio, Seongnam, South Korea). MF-hCADM1 peptide-conjugated BSA was immobilized on a research-grade carboxylic (COOH) sensor chip using an amine coupling kit (Icluebio, Seongnam, South Korea) according to the manufacturer’s instruction. Then, the increasing concentrations (8 nM, 16 nM, 32 nM, 64 nM, and 128 nM) of the selected antibodies in a running buffer containing 10 mM of HEPES-buffered saline, pH of 7.4, 2 mM of CaCl_2_, 1 mM of MnCl_2_, 700 mM of NaCl, and 0.005% (*v*/*v*) tween 20 were injected at a flow rate of 50 µL/min at room temperature. For the regeneration of the sensor chips at each cycle, 10 mM glycine-HCl, with a pH of 2.5, was used to remove bound antibodies from the surfaces. The affinity constant (K_D_) was obtained using the iMSPR analysis software.

### 4.9. Flow Cytometry

To evaluate the selected antibody binding to MF-CADM1 on SCLC cells, 1 × 10^6^ of NCI-H69, NCI-H146, and NCI-H187 cells were fixed with 4% (*w*/*v*) paraformaldehyde (PFA), blocked with 1% (*w*/*v*) BSA in PBS, and stained with 20 μg/mL anti-CADM1 scFv-Fcs at 4 °C for 30 min. The cells were then incubated with Alexa Fluor 488-labeled anti-human Fc antibody (1:200; Invitrogen™, Waltham, MA, USA) at 4 °C for 30 min. The samples were analyzed using flow cytometry (Guava easyCyte, Merck Millipore, Burlington, MA, USA). The data were analyzed using FlowJo software (TreeStar, V 10.6, Ashland, OR, USA).

To test the specific binding of K103.3 to MF-CADM1, 1 × 10^6^ NCI-H69 cells were fixed with 4% (*w*/*v*) paraformaldehyde (PFA), blocked with 1% (*w*/*v*) BSA in PBS, and stained with K103.3 pre-incubated with MF-hCADM1 (4 °C for 1 h). The cells were then incubated with Alexa Fluor 488-labeled anti-human Fc antibody (1:200; Invitrogen™) at 4 °C for 30 min. The samples were analyzed using flow cytometry (Guava easyCyte, Merck Millipore, Burlington, MA, USA). The data were analyzed using FlowJo software (TreeStar, V 10.6).

To evaluate the effects of K103.3 on endothelial cell activation, 2 × 10^5^ HUVECs were incubated in the absence or presence of 20 ng/mL hTNFα (Millipore) or 20 μg/mL K103.3 for 24 h. After blocking with 1% (*w*/*v*) BSA in PBS at room temperature for 1 h, cells were incubated with 2 μg of anti-ICAM-1 (Abcam) or anti-VCAM-1 antibody (Abcam) at 37 °C for 1 h and then with Alexa 647-labeled anti-rabbit IgG antibody (1:1000; Invitrogen™) at 37 °C for 1 h. The samples were analyzed using flow cytometry (Guava easyCyte). The data were analyzed using FlowJo software (TreeStar, V 10.6).

To investigate the expression of CADM1 and MF-CADM1 on HUVECs, 1 × 10^6^ HUVEC cells were fixed with 4% (*w*/*v*) paraformaldehyde (PFA), blocked with 1% (*w*/*v*) BSA in PBS, and stained with 20 μg/mL anti-CADM1 scFv-Fc or chicken anti-CADM1 mAb (1:1000; MBL) at 4 °C for 30 min. The cells were then incubated with Alexa Fluor 488-labeled anti-human Fc antibody (1:200; Invitrogen™) or Alexa Fluor 488-labeled anti-chicken IgY antibody (1:200; Jackson ImmunoResearch Laboratories, West Grove, PA, USA) at 4 °C for 30 min. The samples were analyzed using flow cytometry (Guava easyCyte, Merck Millipore). The data were analyzed using FlowJo software (TreeStar, V 10.6).

### 4.10. T Cell-Mediated Tumor Cell Killing Assay

The 96-well plates were coated with poly-L-lysine at 37 °C for 2 h and washed with PBS three times. After drying, 5 × 10^3^ of the indicated SCLC cells and HUVECs were seeded and incubated at 37 °C overnight. Then, 5 × 10^4^ Jurkat T cells activated by 1 μg/mL anti-CD3ε antibody were incubated with the target cells in 96-well clear flat bottom (Falcon, Corning, NY, USA) in the presence or absence of the indicated concentration of the selected antibodies. Simultaneously, Annexin V red reagent (Sartorius, Goettingen, Germany) was also added to this experimental setting to monitor the death of SCLC cells and HUVECs, which was measured using the IncuCyte^®^ system (Sartorius) hourly for 2 days.

### 4.11. Cell Viability Assay

To test the cell viability of HUVECs in the absence or presence of the selected antibody, 5 × 10^3^ HUVECs were seeded on 96-well plates after cell counting with ADAM-CellT (NanoEntek, Seoul, Korea) and incubated in EGM-2 in the absence or presence of 20 μg/mL of K103.3 or 36 μg/mL of 5-fluorouracil (5-FU) for 24 h at 37 °C. Cell viability was determined using the Cell Counting Kit-8 (Sigma-Aldrich, St Louis, MO, USA) following the manufacturer’s instructions and measured at 450 nm absorbance using a microtiter plate reader (Bio-Tek Instruments).

### 4.12. Statistical Methods

The statistical analyses were carried out using GraphPad Prism (GraphPad Software Inc., San Diego, CA, USA) using the two-tailed Student t-test for comparisons between two groups. *p*-values of < 0.05 were considered statistically significant (* *p* < 0.05; ** *p* < 0.01; *** *p* < 0.001). Graphs are presented as the mean of independent experiments ± standard error of the means (SEM).

## Figures and Tables

**Table 1:** SPR analysis of the binding affinities of the selected scFv-Fc clones to MF-hCADM1.

Antibody (scFv-Fc)	K_D_ (M)	K_on_ (1/M∙s)	K_off_ (1/s)
K103.1	4.49 × 10^−9^	4.63 × 10^4^	2.07 × 10^−4^
K103.2	3.91 × 10^−8^	5.04 × 10^4^	1.97 × 10^−3^
K103.3	2.25 × 10^−9^	1.38 × 10^5^	3.11 × 10^−4^
K103.4	4.12 × 10^−8^	4.59 × 10^4^	1.89 × 10^−3^

## Data Availability

The collected data in this study are available from the corresponding author on reasonable request. The information of the antibody clone sequences is not publicly available due to patent issue.

## Supplementary Materials

The following supporting information can be downloaded at https://www.mdpi.com/article/10.3390/ijms23136895/s1.
