# Supplementary material for: A Fully-Human Antibody Specifically Targeting a Membrane-Bound Fragment of CADM1 Potentiates the T Cell-Mediated Death of Human Small-Cell Lung Cancer Cells
Source: Int J Mol Sci. 2022 Jun 21;23(13):6895. doi: 10.3390/ijms23136895 (PMC9266846; doi:10.3390/ijms23136895)
Supplement: Supplementary file 1 [file ijms-23-06895-s001.zip › ijms-1716775-supplementary.pdf]

A Fully-human Antibody Specifically Targeting a Membrane-bound Fragment of CADM1 Potentiates the T cell-mediated Death of Human Small Cell Lung Cancer Cells

Ji Hyun Lee [1], Ji Woong Kim [2], Ha Rim Yang [1], Seong-Won Song [3], Song-Jae Lee [3], Yeongha Jeon [3], Anna Ju [3], Narim Lee [3], Min-gu Kim [3], Minjoo Kim [3], Kyusang Hwang [1], Jin Hwan Yoon [1], Hyunbo Shim [6] and Sukmook Lee [1,2,4,5,*]

[1] Department of Biopharmaceutical Chemistry, Kookmin University, Seoul 02707, Republic of Korea; 707jh@kookmin.ac.kr (J.H.L); 2825760@kookmin.ac.kr (H.R.Y); kyusang@kookmin.ac.kr (K.H); yoonjinhwan8090@kookmin.ac.kr (J.H.Y)

[2] Department of Chemistry, Kookmin University, Seoul 02707, Republic of Korea; jwk7853@kookmin.ac.kr (J.W.K)

[3] R&D Center, CellabMED Inc., 161, Jeongneung-ro, Seongbuk-gu, Seoul, Republic of Korea; swsong@cellabmed.com (S-W.S), sjlee@cellabmed.com (S-J.L), yeongha0820@cellabmed.com (Y.H.J), anna@cellabmed.com (A.N.J), narim.lee@cellabmed.com (N.R.L), alsrn0520@cellabmed.com (M-G.K), delijoo@cellabmed.com (M.J.K)

[4] Biopharmaceutical Chemistry Major, School of Applied Chemistry, Kookmin University, Seoul 02707, Republic of Korea

[5] Antibody Research Institute, Kookmin University, Seoul 02707, Republic of Korea

[6] Department of Life Science, Ewha Womans University, Seoul 03760, Republic of Korea; hshim@ewha.ac.kr (H.S)

* Correspondence: Lees2018@kookmin.ac.kr; Tel.: +82-2-910-6763

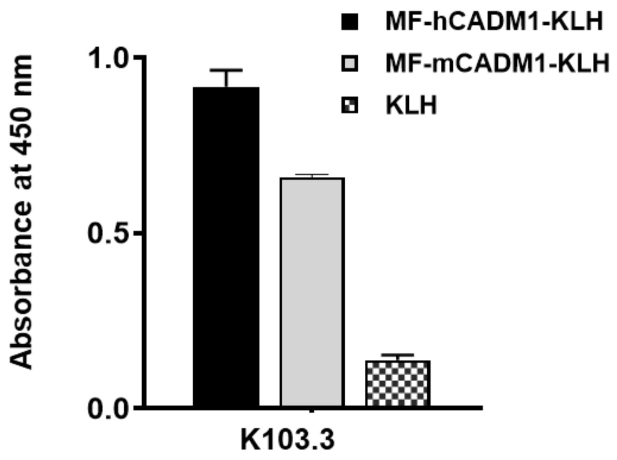

Supplementary Figure S1. ELISA measurement of K103.3 binding to KLH-conjugated human and mouse MF-CADM1. ELISA was performed with MF-hCADM1-KLH or MF-mCADM1-KLH to verify the specific binding of the selected antibody to MF-CADM1. KLH alone was used as a negative control.

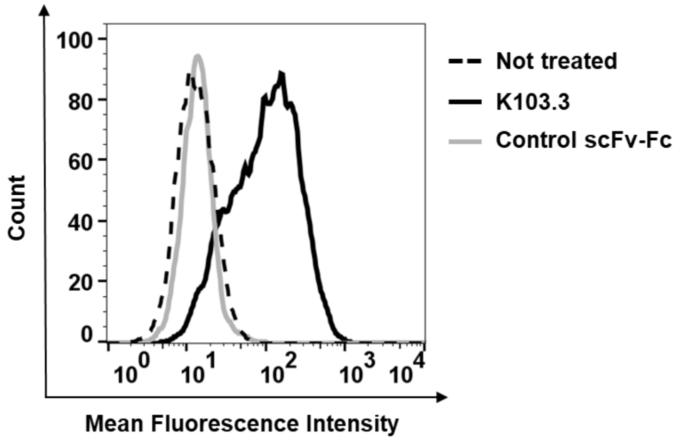

Supplementary Figure S2. Flow cytometry analysis of the selected antibody binding to MF-CADM1 on NCI-H69 cells. Binding of the selected antibody to MF-CADM1 on NCI-H69 cells was investigated in the absence (black dashed line) or presence (black solid line) of K103.3 or control scFv-Fc (gray solid line) using flow cytometry.

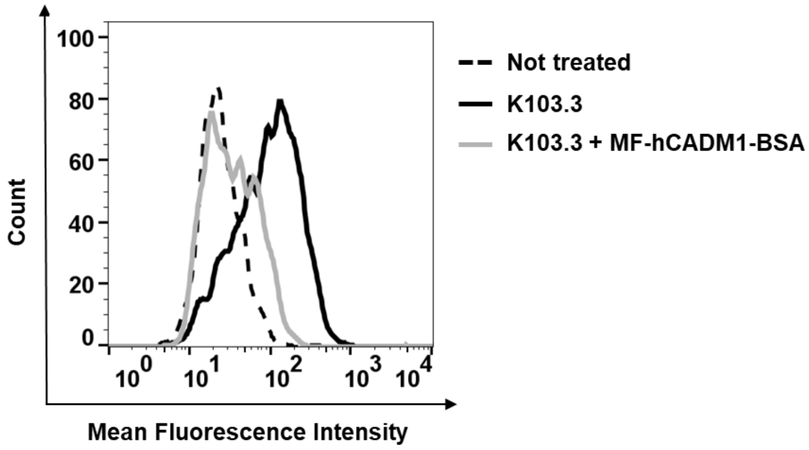

Supplementary Figure S3. The specific binding of the selected antibody to MF-CADM1. Binding of the selected antibody to MF-CADM1 on NCI-H69 cells was investigated in the absence (black dashed line) or presence (black solid line) of K103.3, or pre-incubated with MF-hCADM1-BSA (K103.3 + MF-hCADM1-BSA; gray solid line) using flow cytometry.

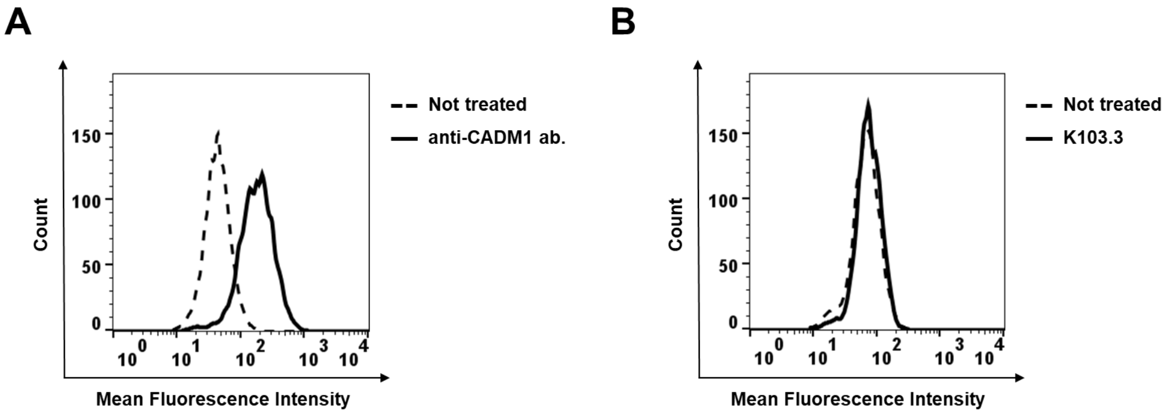

Supplementary Figure S4. Measurement of CADM1 and MF-CADM1 expression on HUVECs. Expression of CADM1 and MF-CADM1 on HUVECs was investigated in the absence (black dashed line) or presence (black solid line) of (A) commercially available CADM1 antibody and (B) K103.3, an MF-CADM1 specific antibody generated in our laboratory, using flow cytometry.

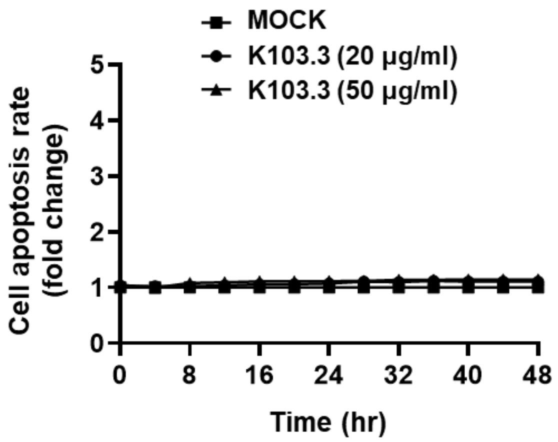

Supplementary Figure S5. Effect of the selected antibody on Jurkat T cell-mediated HUVEC death. Antibody concentration-dependent HUVEC death by Jurkat T cells in the presence or absence of the K103.3 selected antibody was measured with Annexin V.
